# Development of nomogram for predicting major complications in patients with esophageal cancer in the early postoperative period

**DOI:** 10.1186/s12893-023-02090-8

**Published:** 2023-06-29

**Authors:** Maimaiti Mijiti, Dan Li, Rui Yan, Tingting Yuan, Guimei Shen, Dan Zhao

**Affiliations:** The 3rd Affiliated Teaching Hospital of Xinjiang Medical University (Affiliated Cancer Hospital), Urumqi, China

**Keywords:** Esophageal cancer, Complication, Prognostic nutritional index, Prediction model

## Abstract

**Purpose:**

To investigate the predictive value of the Prognostic Nutrition Index (PNI) in major complications after esophagectomy for esophageal cancer and to develop a Nomogram risk prediction model.

**Method:**

The clinical data of 386 patients who underwent radical esophageal cancer surgery from May 2019 to March 2022 were retrospectively analyzed. Logistic regression analysis was performed to screen independent risk factors associated with major postoperative complications. A nomogram risk prediction model for major postoperative complications was developed based on the predictors, and the clinical utility of the model was assessed by decision curve analysis(DCA).

**Result:**

In this study logistic univariate regression analysis found that age, preoperative radiotherapy, American Society of Anesthesiologists physical status (ASA score), length of surgery, and PNI may be associated with the development of major postoperative complications. logistic multifactorial analysis showed that the above risk factors were independent risk factors for the development of major postoperative complications in esophageal cancer. Nomogram was developed by incorporating the above risk factors with ASA classification. The calibration curves showed that the model had a good agreement. The decision curves showed that the model has good clinical application.

**Conclusion:**

Individualized nomograms based on PNI combined with clinical indicators can be used to predict major complications in the early postoperative period and help to enhance perioperative management.

## Background

Esophageal cancer is one of the world's most common causes of cancer death [1]. Currently, the main treatment for esophageal cancer is still a combination of surgery-based therapy. The poor prognosis among many types of surgery has improved recently due to the significant advancement in medical technology [2], although the prevalence of postoperative problems following esophageal cancer resection is still considerable [3]. The incidence rate could be as higher as 65% [4]. According to studies, postoperative problems in patients with esophageal cancer are linked to a poor both short- and long-term prognosis [3, 5, 6]. Postoperative major complications can extend a patient's hospital stay and add to the patient's financial burden and the burden of healthcare professionals [7]. It is crucial to identify patients with potential postoperative problems early to enhance patient prognosis and lessen the burden.

The Prognostic Nutrition Index (PNI) was initially used to predict postoperative complications and morbidity in patients with gastrointestinal tumors. It is calculated from serum albumin and peripheral blood lymphocytes. Serum albumin is a common clinical indicator of the body's nutritional status. Lymphocytes, a type of white blood cell, have a specific immune function and are an indicator of the body's inflammatory state. An increasing number of studies show that inflammatory response and nutritional status are closely related to tumorigenesis, progression, metastasis, and prognosis of tumor patients [8, 9]. Malnutrition may lead to a decrease in the immune function of patients, which in turn worsens their prognosis [10]. Studies have shown the prognostic, predictive value of preoperative PNI in assessing the prognosis of gastrointestinal malignancies such as gastric, intestinal, and hepatic cancers [11–13]. Whether PNI can be used to predict major postoperative complications in patients with esophageal cancer has rarely been reported in the literature. This study aims to investigate the clinical value of PNI in predicting early postoperative major complications in patients with esophageal cancer. It will combine preoperative and intraoperative factors to create a new nomogram to predict major postoperative complications for the treatment and improved prognosis of patients with esophageal cancer.

## Materials and methods

### Patients

We retrospectively analyzed the demographic and clinical data of 386 patients who underwent esophageal cancer resection in our surgical department between May 2019 and March 2022. Inclusion criteria: 1. patients with a precise histological diagnosis of esophageal cancer; 2. undergoing radical esophageal cancer resection; 3. laboratory indices available before and within seven days after surgery. Exclusion criteria: 1. patients with the preoperative combination of severe cardiovascular and cerebrovascular diseases, severe infections, and autoimmune diseases; 2. patients who were lost to follow-up within 30 days after surgery. This study was conducted according to the Declaration of Helsinki. Informed consent was waived by the Ethics Committee of the 3rd Affiliated Teaching Hospital of Xinjiang Medical University(Affiliated cancer Hospital) because the clinical data in the current study were retrospective and anonymous. This study was approved by the Ethics Committee of the 3rd Affiliated Teaching Hospital of Xinjiang Medical University(Affiliated cancer Hospital).

### Variables

Based on previous research experience, we collected the following potential variables associated with postoperative complications, including gender, age, body mass index (BMI), history of smoking, history of alcohol consumption, white blood cell count, red blood cell count, hemoglobin, lymphocyte count, neutrophil count, monocyte count, preoperative chemotherapy before surgery, preoperative radiotherapy, ASA classification, T stage, N stage, TNM stage, duration of surgery, neutrophil–lymphocyte ratio (NLR), platelet-lymphocyte ratio (PLR), monocyte-lymphocyte ratio (MLR), albumin-platelet ratio (APR), and prognostic nutritional index (PNI) [PNI = serum albumin level (g/L) + 5 × peripheral blood lymphocyte count (× 109/L)]. All laboratory indices were collected within seven days before surgery.

### Definition of complications

In this study, postoperative complications were defined as adverse events occurring within 30 days after surgery or during hospitalization, including respiratory, digestive, cardiovascular, neurological, and urinary system-related complications or technical complications, and severity was assessed using the Clavien-Dindo classification. Clavien-Dindo grade III or higher complications were considered major complications.

### Statistical analysis

Free Statistics software version 1.7.1 was used for the statistical analysis of the data. All normally distributed continuous variables were expressed as mean ± standard deviation, and non-normally distributed continuous variables were expressed as median (interquartile range). Categorical variables were analyzed by chi-square with test or Fisher exact test, and continuous variables were analyzed by independent samples t-test or Mann–Whitney U test. The Youden index was calculated using the subject operating characteristic curve (ROC) for neutrophil–lymphocyte ratio (NLR), platelet-lymphocyte ratio (PLR), monocyte-lymphocyte ratio (MLR), albumin-platelet ratio (APR), and prognostic nutritional index (PNI), and the threshold values were calculated and grouped high-low. Univariate logistic regression analysis was performed to screen for complications-associated risk factors. Independent risk factors associated with major postoperative complications were further derived after multifactorial logistic regression analysis. A nomogram risk prediction model for predicting major postoperative complications was developed based on the independent risk factors associated with major complications. The discrimination and calibration of the model were assessed by area under the curve (AUC) and calibration curve. The clinical utility of the model was evaluated by decision curve analysis (DCA). *P* < 0.05 was considered statistically different, and all tests were two-sided.

## Result

### Patient characteristics

**Fig. 1:**
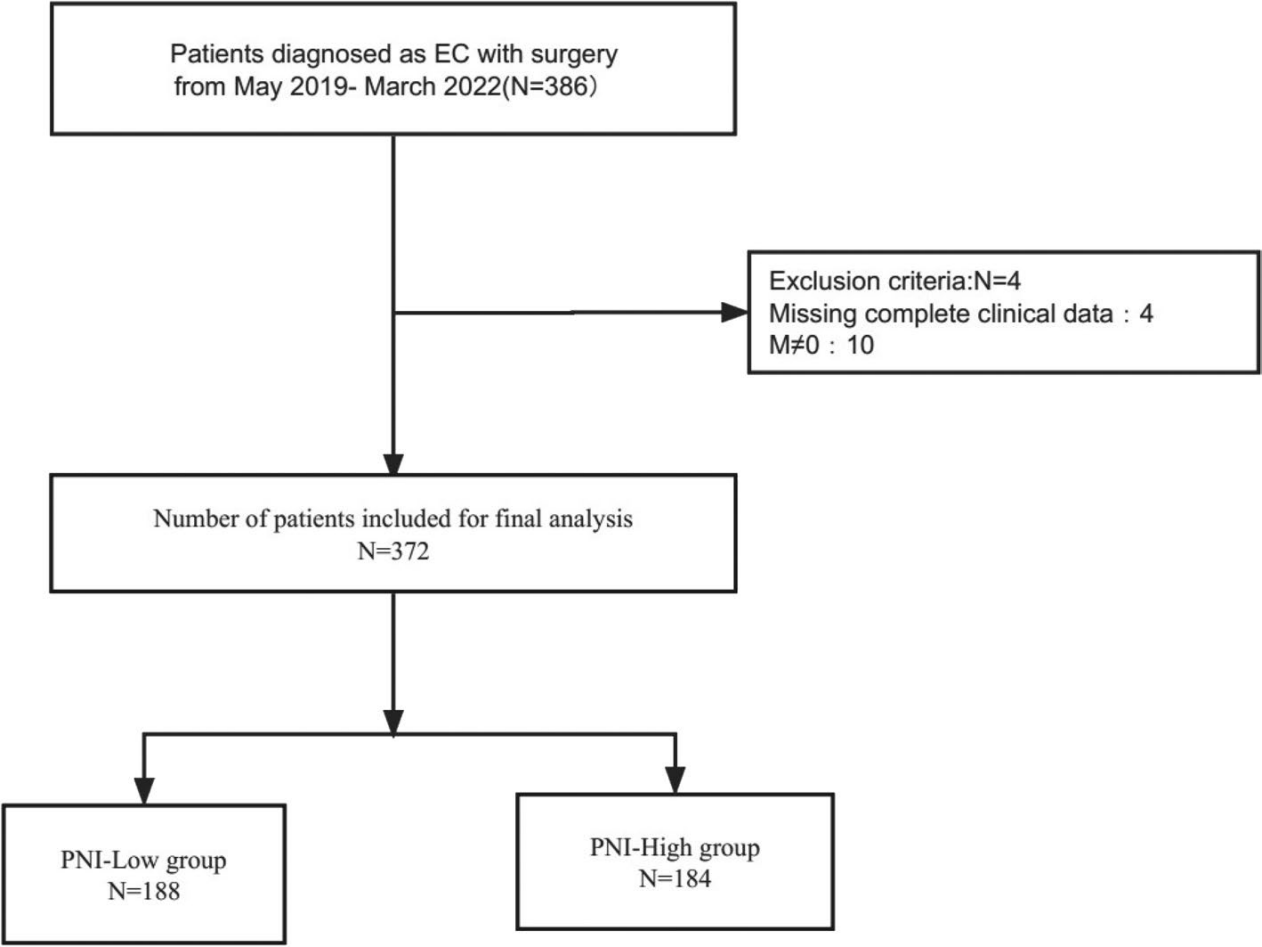
The flow chart of the queue screening process

We retrospectively collected clinical information from 386 patients who underwent esophagectomy between December 2017 and December 2021. Clinical information was missing in 4 cases and 14 cases qualified as exclusion criteria. The final 372 patients were included in the analysis. The inclusion and exclusion process is shown in Fig. 1. 280 (75.3%) of the patients were male, and 121 (32.5%) patients were aged > 60 years. 75 (20.2%) patients had major complications (Table 1).

**Table 1:** Patients’ characteristic

Characteristics	Total (*n *= 372)	PNI-Low Group (< 48.6, *n* = 188)	PNI-High Group (≥ 48.6, *n* = 184)	*P-*value
Gender, n (%)				0.006
Female	92 (24.7)	58 (30.9)	34 (18.5)	
Male	280 (75.3)	130 (69.1)	150 (81.5)	
Age, n (%)				0.071
< 60	121 (32.5)	53 (28.2)	68 (37)	
≥ 60	251 (67.5)	135 (71.8)	116 (63)	
BMI(kg/m^2^), n (%)				0.013
< 24	194 (52.2)	110 (58.5)	84 (45.7)	
≥ 24	178 (47.8)	78 (41.5)	100 (54.3)	
Tobacco use, n (%)				0.017
No	213 (57.3)	119 (63.3)	94 (51.1)	
Yes	159 (42.7)	69 (36.7)	90 (48.9)	
Alcohol use, n (%)				0.298
No	305 (82.0)	158 (84)	147 (79.9)	
Yes	67 (18.0)	30 (16)	37 (20.1)	
RBC(10^12^/L), Mean ± SD	4.5 ± 0.5	4.3 ± 0.5	4.7 ± 0.5	< 0.001
HB(g/L), Mean ± SD	135.5 ± 16.4	128.8 ± 16.1	142.4 ± 13.8	< 0.001
WBC(10^9^ /L), Median (IQR)	6.0 (5.1, 7.0)	5.6 (4.8, 6.8)	6.3 (5.4, 7.2)	< 0.001
NEUT(10^9^ /L), Median (IQR)	3.5 (2.8, 4.3)	3.5 (2.7, 4.4)	3.6 (2.9, 4.3)	0.473
LYM(10^9^ /L), Median (IQR)	1.7 (1.4, 2.1)	1.4 (1.2, 1.7)	2.0 (1.7, 2.4)	< 0.001
MO(10^9^ /L), Median (IQR)	0.4 (0.4, 0.6)	0.4 (0.3, 0.5)	0.5 (0.4, 0.6)	0.042
Preoperative Chemotherapy, n (%)				0.031
No	340 (91.4)	166 (88.3)	174 (94.6)	
Yes	32 ( 8.6)	22 (11.7)	10 (5.4)	
Preoperative Radiotherapy, n (%)				0.004
No	360 (96.8)	177 (94.1)	183 (99.5)	
Yes	12 ( 3.2)	11 (5.9)	1 (0.5)	
ASA, n (%)				0.366
< 3	230 (61.8)	112 (59.6)	118 (64.1)	
≥ 3	142 (38.2)	76 (40.4)	66 (35.9)	
T stage, n (%)				0.295
T0 + T1 + T2	108 (29.0)	50 (26.6)	58 (31.5)	
T3 + T4	264 (71.0)	138 (73.4)	126 (68.5)	
N stage, n (%)				0.729
N0 + N1	274 (73.7)	137 (72.9)	137 (74.5)	
N2 + N3	98 (26.3)	51 (27.1)	47 (25.5)	
TNM stage, n (%)				0.606
0 + I + II	183 (49.2)	90 (47.9)	93 (50.5)	
III + IV	189 (50.8)	98 (52.1)	91 (49.5)	
Location of cancer				0.78
Upper	38(10.2)	20(10.6)	18(9.8)	
Middle	181 (48.7)	94 (50)	87 (47.3)	
Lower	153 (41.1)	74 (39.4)	79 (42.9)	
Histology of cancer				0.864
Squamous cell carcinoma	322 (86.6)	161 (85.6)	161 (87.5)	
Adenocarcinoma	41 (11.0)	22 (11.7)	19 (10.3)	
Other	9 ( 2.4)	5 (2.7)	4 (2.2)	
Surgical method				0.338
Sweet	86 (23.1)	49 (26.1)	37 (20.1)	
Ivor-lewis	260 (69.9)	125 (66.5)	135 (73.4)	
McKeown	26 ( 7.0)	14 (7.4)	12 (6.5)	
Lymph node dissection				0.228
Two-field	201 (54.0)	99 (52.7)	102 (55.4)	
Three-field	171 (46.0)	89 (47.3)	82 (44.6)	
Anastomotic methods				0.159
Cervical anastomosis	252 (67.7)	121 (64.4)	131 (71.2)	
Thoracic anastomosis	120 (32.3)	67 (35.6)	53 (28.8)	
Surgery time (hour), Mean ± SD	5.4 ± 1.6	5.1 ± 1.6	5.6 ± 1.5	0.003
NLR, n (%)				< 0.001
< 1.91	160 (43.0)	56 (29.8)	104 (56.5)	
≥ 1.91	212 (57.0)	132 (70.2)	80 (43.5)	
PLR, n (%)				< 0.001
< 121.01	144 (38.7)	49 (26.1)	95 (51.6)	
≥ 121.01	228 (61.3)	139 (73.9)	89 (48.4)	
MLR, n (%)				< 0.001
< 0.23	153 (41.1)	49 (26.1)	104 (56.5)	
≥ 0.23	219 (58.9)	139 (73.9)	80 (43.5)	
APR, n (%)				0.283
< 0.15	121 (32.5)	66 (35.1)	55 (29.9)	
≥ 0.15	251 (67.5)	122 (64.9)	129 (70.1)	
Calvien Dindo classification, n (%)				0.009
< 3	297 (79.8)	140 (74.5)	157 (85.3)	
≥ 3	75 (20.2)	48 (25.5)	27 (14.7)	

*BMI* Body mass index, *RBC* Red blood cell, *HB* Hemoglobin, *WBC* White blood cell, *NEUT* Neutrophilicgranulocyte, *LYM* Lymphocyte, *MO* Monocytes, *ASA* American Society of Anesthesiologists physical status, *NLR* Neutrophil–lymphocyte ratio, *PLR* Platelet-lymphocyte ratio, *MLR* Monocyte-lymphocyte ratio, *APR* Albumin-platelet ratio, *PNI* Prognostic nutritional index, *SD* StandardDeviation, *IQR* Interquartile range

### Comparison of baseline information between high and low PNI groups

The maximum Youden index (0.295) was calculated from the ROC curve, and the optimal cut-off value of 48.6 was selected, with a sensitivity of 64% and specificity of 53%. Using 48.6 as the cut-off point, PNI ≥ 48.6 was considered as the high PNI group (184 cases) and < 48.6 as the low PNI group (188 topics). Major complications occurred in 48 (25.5%) patients in the low PNI group and 27 (14.7%) patients in the high PNI group, with a higher incidence of major complications in the low PNI group compared to the high PNI group (*p* = 0.009) (Table 1).

### Results of univariate and multivariate logistic regression analysis of risk factors for major postoperative complications

Univariate analysis revealed that age (OR 2.73, 95% CI 1.43–5.18, *p* = 0.002), preoperative radiotherapy (OR 4.22, 95% CI 1.32–13.48), *p* = 0.015), ASA classification (OR 1.91, 95% CI 1.14–3.18, *p* = 0.013), operation time ( OR 1.2, 95% CI 1.02–1.4, *p* = 0.025), and PNI (OR 0.5, 95% CI 0.3–0.85, *p* = 0.01) may be associated with the development of major postoperative complications. Logistic multifactorial analysis adjusting for age, preoperative radiotherapy, ASA classification, and length of surgery showed that age (OR 2.35, 95% CI 1.19–4.67,*p* = 0.02), preoperative radiotherapy (OR 4.57, 95% CI 1.29–16.21, *p* = 0.019), operation time (OR 1.25, 95% CI 1.06- 1.48, *p* = 0.007), and PNI (OR 0.52, 95% CI 0.3–0.9, *p* = 0.02) were independent risk factors for major postoperative complications of esophageal cancer. ASA classification (OR 1.57, 95% CI 0.91–2.72, *p* = 0.103) was not an independent risk factor for major complications. High PNI was a protective factor for major postoperative complications (Table 2).

**Table 2:** Univariate and multivariate logistic regression analysis

Characteristics	Univariable			Multivariable		
	OR	95%CI	*P-*value	adjOR	95%CI	adj *P-*value
Gender						
Female						
Male	0.81	0.46–1.43	0.463			
Age						
< 60						
≥ 60	2.73	1.43–5.18	0.002	2.35	1.19–4.67	0.02
BMI(kg/m^2^)						
< 24						
≥ 24	1.23	0.74–2.04	0.421			
Tobacco use,						
No						
Yes	1.07	0.64–1.78	0.805			
Alcohol use						
No						
Yes	0.94	0.48–1.84	0.864			
RBC(10^12^/L)	0.8	0.5–1.27	0.347			
HB(g/L)	0.99	0.97–1	0.069			
WBC(10^9^ /L)	1.06	0.92–1.2	0.426			
NEUT(10^9^ /L)	1.05	0.91–1.22	0.501			
LYM(10^9^ /L)	0.98	0.8–1.18	0.804			
MO(10^9^ /L)	3.34	0.62–17.87	0.159			
Preoperative Chemotherapy						
No						
Yes	1.92	0.87–4.26	0.107			
Preoperation Radiotherapy						
No						
Yes	4.22	1.32–13.48	0.015	4.57	1.29–16.21	0.019
ASA						
< 3						
≥ 3	1.91	1.14–3.18	0.013	1.57	0.91–2.72	0.103
T stage						
T0 + T1 + T2						
T3 + T4	0.78	0.45–1.33	0.359			
N stage						
N0 + N1						
N2 + N3	1.66	0.96–2.86	0.069			
TNM stage						
0 + I + II						
III + IV	1.21	0.73–2.02	0.455			
Location of cancer						
Upper						
Middle	0.86	0.37–1.96	0.714			
Lower	0.72	0.31–1.69	0.454			
Histology of cancer						
Squamous cell carcinoma						
Adenocarcinoma	0.65	0.26–1.62	0.356			
Other	1.09	0.22–5.36	0.918			
Surgical method						
Sweet						
Ivor-lewis	1.27	0.68–2.39	0.458			
McKeown	1.13	0.37–3.46	0.835			
Lymph node dissection						
Two-field						
Three-field	1.11	0.67–1.84	0.693			
Anastomotic methods						
Cervical anastomosis						
Thoracic anastomosis	1.43	0.84–2.42	0.185			
Surgery time (hour)	1.2	1.02–1.4	0.025	1.25	1.06–1.48	0.007
NLR						
< 1.91						
≥ 1.91	0.83	0.5–1.38	0.475			
PLR						
< 121.01						
≥ 121.01	0.62	0.37–1.03	0.066			
MLR						
< 0.23						
≥ 0.23	1.51	0.89–2.58	0.126			
APR						
< 0.15						
≥ 0.15	0.62	0.37–1.04	0.07			
PNI						
< 48.6						
≥ 48.6	0.5	0.3–0.85	0.01	0.52	0.3–0.9	0.02

*BMI* Body mass index, *RBC* Red blood cell, *HB* Hemoglobin, *WBC* White blood cell, *NEUT* Neutrophilicgranulocyte, *LYM* Lymphocyte, *MO* Monocytes, *ASA* American Society of Anesthesiologists physical status, *NLR* Neutrophil–lymphocyte ratio, *PLR* Platelet-lymphocyte ratio, *MLR* Monocyte-lymphocyte ratio, *APR* Albumin-platelet ratio, *PNI* prognostic nutritional index, *adj P-value* Adjusted *P*-value

### Development and validation of nomogram

**Fig. 2:**
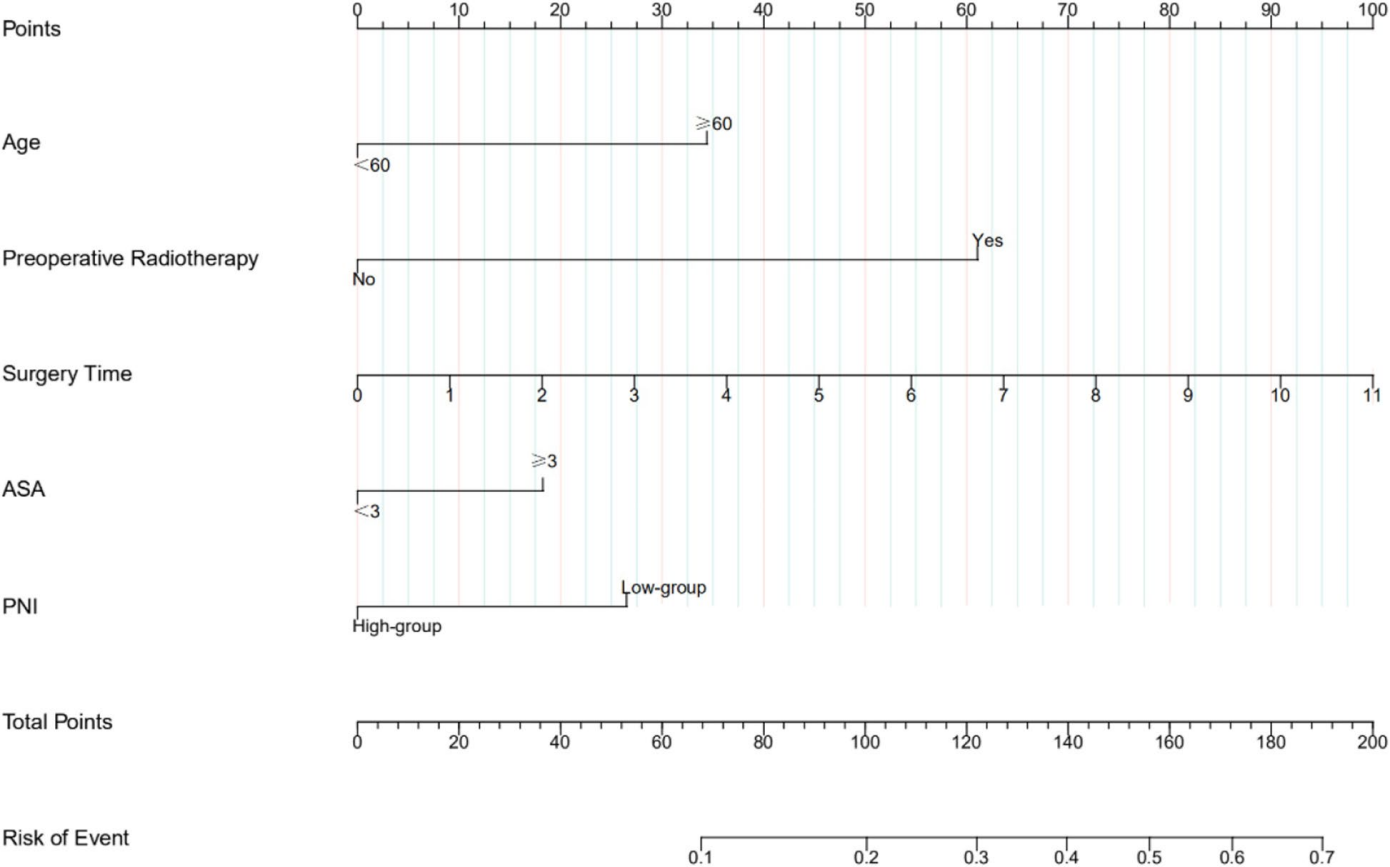
Nomogram for predicting major complications in patients with esophageal cancer

**Fig. 3:**
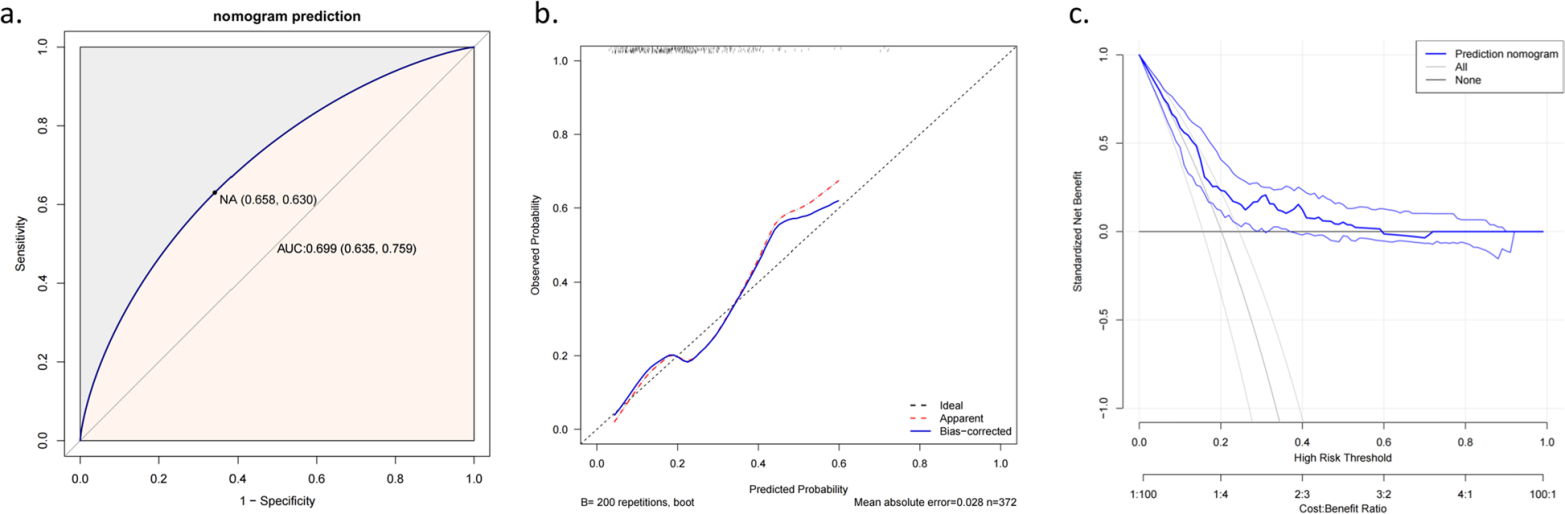
Validation of the nomogram: **a** Receiver operating characteristic curves for risk model to predict the major complications after surgery. **b** Calibration curves for the nomogram to estimate major complications after surgery. **c** Decision curve analysis for the nomogram to estimate major complications after surgery

Based on the multivariate logistic analysis, we established a nomogram by four predictors related to major postoperative complications, including age, preoperative radiotherapy, length of surgery, PNI, and ASA grading (Fig. 2). According to previous studies, ASA grading was closely related to patient prognosis. Hence, the ASA grading was incorporated into the nomogram in conjunction with the clinic. The area under the ROC curve (RAUC) of the model was 0.699, indicating that the model has good discrimination (Fig. 3a). The calibration curve showed a good consistency between the predicted and actual observed values for the occurrence of major complications (Fig. 3b). The clinical benefit of the model was validated using decision curves (DCA). The results showed that the DCA curve of the nomogram showed a positive net benefit over a wide range of threshold probabilities, indicating a better clinical application of the nomogram(Fig. 3c).

In addition, the net reclassification index (NRI) for this nomogram was 0.325 (*p* = 0.011), and the integrated discrimination index (IDI) was 0.056 (*p* < 0.001) compared to TNM staging. This indicates that the ability of this nomogram to predict major postoperative complications was improved compared with TNM staging.

## Discussion

Currently, there are no reliable prediction models for predicting major complications in patients with esophageal cancer. Although several studies have developed nomograms to predict poor prognosis in patients with esophageal cancer, most are limited to predicting postoperative mortality [14–16]. Thus, we retrospectively analyzed the clinical data of 372 patients to determine the predictors of major complications in patients with esophageal cancer in the early postoperative period. The results showed that age, preoperative radiotherapy, length of surgery, and PNI were independent predictors associated with major complications within 30 days after radical esophageal cancer surgery. We analyzed these associated independent predictors and developed and validated a Nomogram.

The specific anatomical site of the esophagus and the difficulty in defining the cancerous part of the esophagus lead to a complex and long-lasting esophagectomy procedure. Besides, those who develop the disease are older and prone to numerous comorbidities, and the incidence of postoperative complications varies widely. In our cohort, complications were evaluated according to the Calvien-Dindo complication grading system, with grade III and above complications considered major complications. The incidence of major complications in our study reached 20.2%, which is consistent with 21.2% in the study of S.J. Davies et al. [17].

Furthermore, advanced age was associated with an increase in postoperative complications. In our study, the incidence of major postoperative complications was significantly increased in patients aged ≥ 60 years. This result was similar to the findings of Qi et al. [18]. Our Multivariable analysis showed that prolonged operative time was an independent risk factor for serious postoperative complications. Operative time > 3 h was not an independent risk factor associated with postoperative complications in the study of Qi [18] et al., whereas operative time was included in our study as a continuous variable in the analysis. There may also be some heterogeneity due to differences in the study area and the level of medical technology development. Our results showed that preoperative radiotherapy was an independent risk factor for serious postoperative complications in patients with esophageal cancer. Patients in our cohort who underwent preoperative radiotherapy involved the early refusal of surgery, advanced age, cardiopulmonary comorbidities, and other reasons requiring radiotherapy followed by selective surgery; in addition, patients often experienced lymphopenia after preoperative radiotherapy, resulting in decreased immune function. Therefore, considering multiple factors, patients in this cohort who underwent preoperative radiotherapy had a higher incidence of postoperative complications and a significantly higher probability of major complications.

The ASA scoring system allows for the rapid classification of patients based on their physical status and comorbidities and can be used for preoperative assessment of morbidity and mortality in surgical patients. Wen-Quan Yu, Gooszen et al. reported that ASA grade ≥ III is an independent risk factor for major complications such as anastomotic fistula [5, 19]. Although ASA grade ≥ III was not statistically significant in multifactorial analysis, this may be due to the heterogeneity of our study. Therefore, we combined our experience in clinical work and included ASA grading in the Nomogram.

Our study demonstrates that low PNI is associated with an increased incidence of major postoperative complications. The mechanism of the correlation between PNI and postoperative complications in patients with esophageal cancer is unclear and may be determined by the role of albumin and lymphocytes. Albumin is the most abundant protein in human serum, and its concentration not only reflects the nutritional status of the body and participates in maintaining the body's immune function but also can stabilize cell growth and DNA replication and plays an essential role in antioxidant, anti-inflammatory, and apoptosis prevention [20]. Studies have confirmed that low preoperative protein levels are associated with poor prognosis in patients with gastrointestinal malignancies [21]. And lymphocytes reflect the cell-mediated immune response. Tumor-infiltrating lymphocytes in the tumor microenvironment play an important role in anti-tumor immunity. A decrease in lymphocytes reduces the anti-tumor response of lymphocytes, creating an environment of low lymphocyte infiltration suitable for tumor cell infiltration and metastasis, predicting a more infiltrative tumor [22]. Therefore, the low PNI status of patients may provide a favorable microenvironment for this immunosuppressed state.

PNI is a simple, non-invasive, and easily accessible index. Accordingly, we developed a new nomogram based on PNI to predict major complications in patients with esophageal cancer in the early postoperative period. Based on multivariate analysis and the results of previous studies, we developed the new nomogram by including five variables, including age, preoperative radiotherapy, ASA classification, duration of surgery, and PNI. We evaluated the performance of the nomogram using ROC curves and calibration curves. The area under the ROC curve (AUC) for major complications was 0.699, demonstrating good accuracy. The calibration curve for the probability of major postoperative complications showed good agreement between the nomogram's predicted and actual observed values. The DCA curve was applied to assess the net benefit of the nomogram for patients. The DCA curve showed that the nomogram resulted in a positive net benefit for patients within a threshold probability range of 0.05–0.60. In addition, the NRI showed that the predictive accuracy of this nomogram was better than that of TNM staging (NRI > 0). IDI showed an improvement in the accuracy of the nomogram in predicting major complications compared with TNM staging. In conclusion, the above validation methods showed that the newly constructed column line graph model had the better net benefit and predictive accuracy. This column line graph is the first time to predict major postoperative complications in patients with esophageal cancer by PNI. In clinical practice, it may help to identify patients at high risk of major postoperative complications at an early stage, which is beneficial for clinicians to target individualized treatment plans, establish perioperative early warning mechanisms, intervene, diagnose and treat early, prevent and reduce the incidence of major postoperative complications in patients, and thus provide patients with better clinical services and medical benefits.

The following limitations exist in this study: 1. This is a retrospective study, and selection bias may exist, so we trained our investigators and included the study population strictly according to the nadir criteria to minimize selection bias. 2. There is no uniform standard for the optimal PNI cut-off value; in this study, the ROC curve determined the optimal PNI cut-off value. However, our PNI cut-off value was similar to Qi [18] et al. (48.6 vs. 48.33). 3. The follow-up period in this study was only 30 days postoperatively, and there was a lack of long-term follow-up information, and we would conduct further long-term follow-up in the future, which may lead to different results. 4. The clinical data in this cohort were obtained from a single medical institution, and we intend to conduct further prospective studies with large samples for external validation to confirm our results.

In conclusion, PNI is an essential predictor of postoperative complications. Although PNI is associated with postoperative complications, previous studies have not included PNI in nomograms to develop predictive models. We created a simple and practical column line chart based on PNI, which has good predictive power for major postoperative complications in patients with esophageal cancer. In clinical practice, patients can be risk-stratified according to our Nomogram to provide early nutritional support and enhance perioperative management and monitoring in high-risk patients for maximum benefit.

## Data Availability

All data generated or analysed during this study are included in this published article.

## References

[1] Morgan E, Soerjomataram I, Rumgay H, Coleman HG, Thrift AP, Vignat J (2022). The Global Landscape of Esophageal Squamous Cell Carcinoma and Esophageal Adenocarcinoma Incidence and Mortality in 2020 and Projections to 2040: New Estimates From GLOBOCAN 2020. Gastroenterology.

[2] Waters JK, Reznik SI (2022). Update on Management of Squamous Cell Esophageal Cancer. Curr Oncol Rep.

[3] Xu QL, Li H, Zhu YJ, Xu G (2020). The treatments and postoperative complications of esophageal cancer: a review. J Cardiothorac Surg.

[4] van der Werf LR, Busweiler LAD, van Sandick JW, van Berge Henegouwen MI, Wijnhoven BPL (2020). Dutch Upper GICAg: Reporting National Outcomes After Esophagectomy and Gastrectomy According to the Esophageal Complications Consensus Group (ECCG). Ann Surg.

[5] Yu WQ, Gao HJ, Shi GD, Tang JY, Wang HF, Hu SY (2021). Development and validation of a nomogram to predict anastomotic leakage after esophagectomy for esophageal carcinoma. J Thorac Dis.

[6] Kauppila JH, Johar A, Lagergren P (2020). Postoperative Complications and Health-related Quality of Life 10 Years After Esophageal Cancer Surgery. Ann Surg.

[7] Goense L, van Dijk WA, Govaert JA, van Rossum PS, Ruurda JP, van Hillegersberg R (2017). Hospital costs of complications after esophagectomy for cancer. Eur J Surg Oncol.

[8] Ye J, Liao B, Jiang X, Dong Z, Hu S, Liu Y (2020). Prognosis Value of Platelet Counts, Albumin and Neutrophil-Lymphocyte Ratio of Locoregional Recurrence in Patients with Operable Head and Neck Squamous Cell Carcinoma. Cancer Manag Res.

[9] Zitvogel L, Pietrocola F, Kroemer G (2017). Nutrition, inflammation and cancer. Nat Immunol.

[10] Collins N, Belkaid Y (2022). Control of immunity via nutritional interventions. Immunity.

[11] Tamai M, Kiuchi J, Kuriu Y, Arita T, Shimizu H, Ohashi T (2021). Clinical impact of postoperative prognostic nutritional index in colorectal cancer patients undergoing adjuvant chemotherapy. Am J Cancer Res.

[12] Li Q, Chen C, Zhang J, Wu H, Qiu Y, Song T (2021). Prediction Efficacy of Prognostic Nutritional Index and Albumin-Bilirubin Grade in Patients With Intrahepatic Cholangiocarcinoma After Radical Resection: A Multi-Institutional Analysis of 535 Patients. Front Oncol.

[13] Sugawara K, Yamashita H, Urabe M, Okumura Y, Yagi K, Aikou S (2020). Poor nutritional status and sarcopenia influences survival outcomes in gastric carcinoma patients undergoing radical surgery. Eur J Surg Oncol.

[14] Yoon JP, Nam JS, Abidin M, Kim SO, Lee EH, Choi IC, et al. Comparison of Preoperative Nutritional Indexes for Outcomes after Primary Esophageal Surgery for Esophageal Squamous Cell Carcinoma. Nutrients. 2021;13(11):4086. 10.3390/nu13114086 PMC8619324 34836339

[15] Okadome K, Baba Y, Yagi T, Kiyozumi Y, Ishimoto T, Iwatsuki M (2020). Prognostic Nutritional Index, Tumor-infiltrating Lymphocytes, and Prognosis in Patients with Esophageal Cancer. Ann Surg.

[16] Zhang H, Shang X, Ren P, Gong L, Ahmed A, Ma Z (2019). The predictive value of a preoperative systemic immune-inflammation index and prognostic nutritional index in patients with esophageal squamous cell carcinoma. J Cell Physiol.

[17] Davies SJ, West MA, Rahman SA, Underwood TJ, Marino LV (2021). Oesophageal cancer: The effect of early nutrition support on clinical outcomes. Clin Nutr ESPEN.

[18] Qi Q, Song Q, Cheng Y, Wang N (2021). Prognostic Significance of Preoperative Prognostic Nutritional Index for Overall Survival and Postoperative Complications in Esophageal Cancer Patients. Cancer Manag Res.

[19] Gooszen JAH, Goense L, Gisbertz SS, Ruurda JP, van Hillegersberg R, van Berge Henegouwen MI (2018). Intrathoracic versus cervical anastomosis and predictors of anastomotic leakage after oesophagectomy for cancer. Br J Surg.

[20] Nazha B, Moussaly E, Zaarour M, Weerasinghe C, Azab B (2015). Hypoalbuminemia in colorectal cancer prognosis: Nutritional marker or inflammatory surrogate?. World J Gastrointest Surg.

[21] He H, Ma Y, Zheng Z, Deng X, Zhu J, Wang Y (2022). Early versus delayed oral feeding after gastrectomy for gastric cancer: a systematic review and meta-analysis. Int J Nurs Stud.

[22] Chou C, Li MO (2018). Re(de)fining Innate Lymphocyte Lineages in the Face of Cancer. Cancer Immunol Res.

