# Correction: Development of nomogram for predicting major complications in patients with esophageal cancer in the early postoperative period

**DOI:** 10.1186/s12893-023-02277-z

**Published:** 2023-12-11

**Authors:** Maimaiti Mijiti, Dan Li, Rui Yan, Tingting Yuan, Guimei Shen, Dan Zhao

**Affiliations:** The 3rd Affiliated Teaching Hospital of Xinjiang Medical University (Affiliated Cancer Hospital), Urumqi, China

**Correction to: BMC Surgery (2023) 23:1**

10.1186/s12893-023-02090-8

Following publication of the original article [1], in Fig. 1 there is a small error; the figure should have appeared as shown below.

**Fig. 1:**
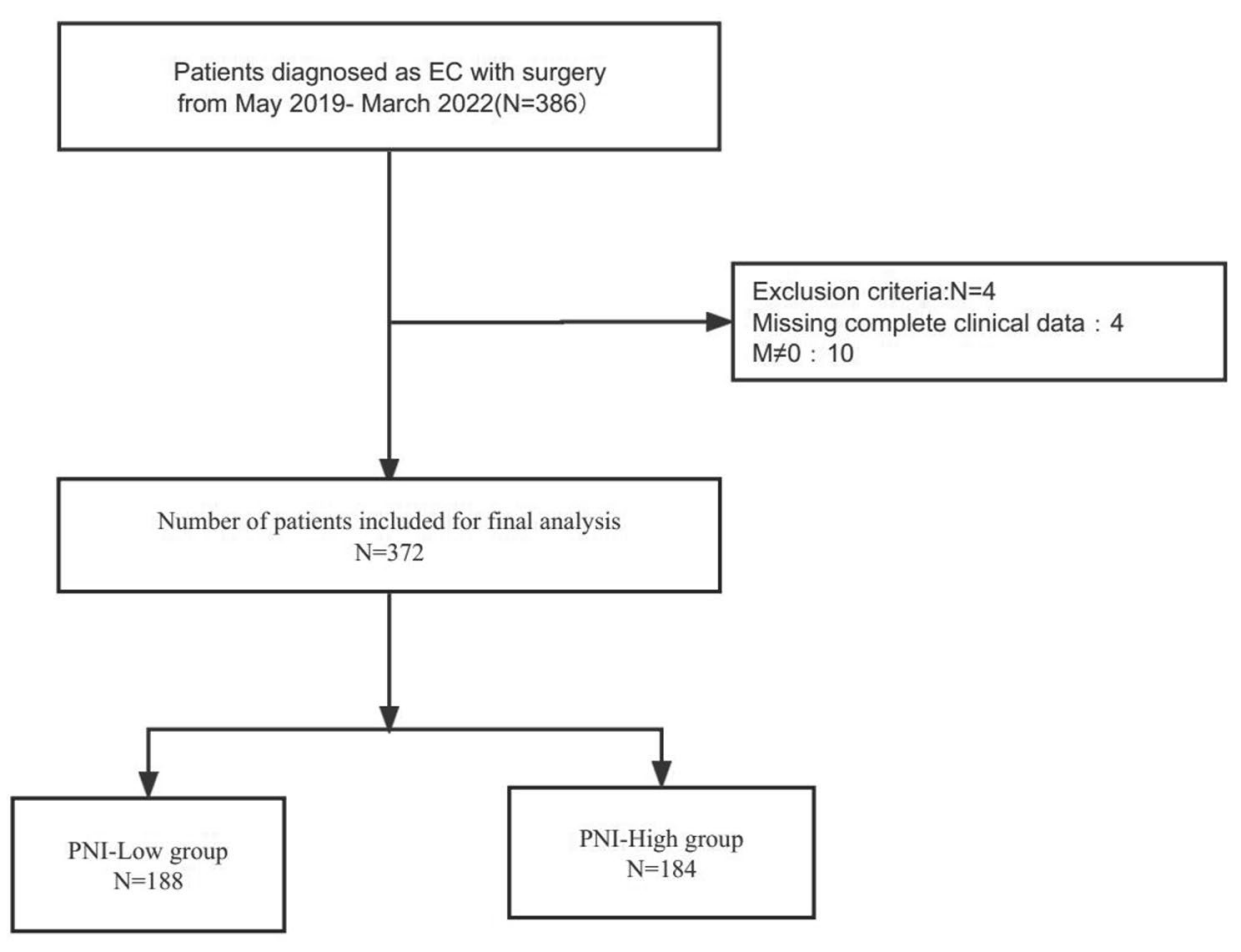
The flow chart of the queue screening process

The original article has been corrected.

